# Whole-exome sequencing in pediatric patients with glomerulonephritis

**DOI:** 10.3389/fgene.2025.1611340

**Published:** 2025-09-12

**Authors:** Marina Peric, Marija Brankovic, Natasa Stajic, Jovana Putnik, Aleksandra Paripovic, Milena Jankovic, Dejan Nikolic, Filip Milanovic, Ivana Novakovic, Vladislav Vukomanovic

**Affiliations:** ^1^ Institute for Mother and Child Healthcare of Serbia “Dr. Vukan Cupic”, Belgrade, Serbia; ^2^ Department for Clinical Genetics, University Children’s Hospital, Belgrade, Serbia; ^3^ University of Belgrade - Faculty of Medicine, Belgrade, Serbia; ^4^ Neurology Clinic, University Clinical Center of Serbia, Belgrade, Serbia; ^5^ Department of Physical Medicine and Rehabilitation, University Children’s Hospital, Belgrade, Serbia; ^6^ Pediatric Surgery Department, University Children’s Hospital, Belgrade, Serbia; ^7^ Institute for Human Genetics, University of Belgrade - Faculty of Medicine, Belgrade, Serbia

**Keywords:** glomerulonephritis, whole-exome sequencing, Alport syndrome, atypical hemolytic uremic syndrome, systemic lupus erythematosus, Henoch–Schoenlein purpura

## Abstract

**Introduction:**

High-throughput sequencing methods revealed disease-causing and susceptibility genes underlying glomerulonephritis (GN). Genetic disorders mimicking GN may be diagnosed in this way. The aim of this study was to perform whole-exome sequencing (WES) in a cohort of sporadic pediatric patients diagnosed with primary or secondary GN.

**Method:**

Thirty-one patients with GN and 50 nephrologically and immunologically healthy pediatric patients (control group - CG) were genetically analyzed. Allele frequencies were compared with the GnomAD database. WES was performed in the laboratory 3billion in South Korea.

**Results:**

Among 10 patients with primary GN, two patients were positive on WES (20%). One had a likely pathogenic heterozygous variant in the *COL4A3* gene associated with Alport syndrome, and one had a heterozygous novel variant of uncertain significance in the *CD46* gene associated with atypical hemolytic uremic syndrome (aHUS). In two of 14 patients with systemic lupus erythematosus (SLE) and GN, a heterozygous pathogenic variant (c.841_849 + 19del) in the *C2* gene was detected. We found no significant variants in seven patients with Henoch–Schönlein purpura (HSP) and GN.

**Conclusion:**

WES helped us detect hereditary diseases that have a clinical presentation like GN, including Alport syndrome and possible aHUS. Finding susceptibility genes in GN helped us understand disease pathophysiology.

## 1 Introduction

Glomerulonephritis (GN) is a heterogeneous group of inflammatory diseases that affect renal glomeruli (Rodríguez-Iturbe et al., 2016). Primary GNs affect only the kidneys and are divided into several main subtypes: immunoglobulin A nephropathy (IgAN), membranous nephropathy (MN), minimal change disease (MCD), focal segmental glomerulosclerosis (FSGS), membranoproliferative GN (MPGN), and rare forms such as steroid-sensitive nephrotic syndrome (SSNS) and anti-glomerular basement membrane (anti-GBM) disease. In secondary GNs, kidneys are damaged due to the presence of another systemic disease, most commonly systemic lupus erythematosus (SLE) and Henoch–Schoenlein purpura (HSP) (Rodríguez-Iturbe et al., 2016; Floege and Amann, 2016; Floege, 2013).

High-throughput sequencing methods, such as genome-wide association studies (GWAS), whole-exome sequencing (WES), and whole-genome sequencing (WGS), revealed disease-causing and susceptibility genes underlying primary and secondary GNs. These methods have contributed to the understanding of genetic background and pathogenetic mechanisms of these complex diseases, which subsequently leads to the development of noninvasive genetic screening, new diagnostic methods, and drug interventions (Floege and Amann, 2016; Wu et al., 2022). IgA nephropathy is associated mostly with the genes encoding proteins of the immune system (Floege, 2013; Wu et al., 2022; Jiyun et al., 2012; Yang et al., 2012). In MN, the podocyte gene PLA2R1 is also of interest (Wu et al., 2022; Stanescu et al., 2011; Yoshikawa and Asaba, 2020), while FSGS is mostly related to the structural genes of the kidney cells (Wu et al., 2022). HLA polymorphisms are genetic factors associated with lupus nephritis (LN) and HSP nephritis (Huang et al., 2023; López-Mejías et al., 2015; Jiang et al., 2017; Koskela et al., 2021). Genes important for interferon signaling are also associated with LN (Huang et al., 2023).

In addition, some genetic disorders may mimic GNs. Those disorders must be recognized to treat these patients adequately, to understand disease prognosis, and to give genetic advice. Among these disorders are Alport syndrome, congenital nephrotic syndromes, atypical hemolytic uremic syndrome (aHUS), etc. (Rahimzadeh et al., 2023; Hao et al., 2020).

The aim of this study was to investigate the genetic backgrounds of a cohort of consecutive sporadic pediatric patients diagnosed with primary or secondary glomerulonephritis using whole-exome sequencing analysis. The results may have important implications for understanding the genetic epidemiology of GN in Serbia and the whole region.

## 2 Methods

### 2.1 Patients

This research is a cross-sectional study approved by the Ethics Committee of the Faculty of Medicine, University of Belgrade (No. 1322/VII-48 from July 29, 2020). The research was conducted at the Institute for Mother and Child Healthcare of Serbia “Dr. Vukan Cupic” from March 1, 2021, to August 1, 2021. Written informed consent has been obtained from all patients or their parents/caregivers if the patient was younger than 15 years.

Patients with primary and secondary GNs due to SLE and HSP were consecutively collected from the inpatient and outpatient units of the institute. Diagnosis was based on the clinical presentation, laboratory results, and, in most cases, histopathology findings. Patients with non-inflammatory glomerular disease (including several children with diabetes mellitus and several children with non-steroidal anti-inflammatory drug use); patients with a positive family history of kidney disease or proven genetic kidney diseases; and those who have GN but with the presence of another significant disease unrelated to GN were excluded.

A total of 43 patients were collected, of which two were excluded due to parents’ refusal to allow their child to participate in the study. Two additional patients were excluded due to the presence of anti-neutrophilic cytoplasmic autoantibody (ANCA) vasculitis with GN. Eight patients had other autoimmune diseases (SLE and HSP) and were suspected of having GN but were not found to have any kidney involvement, so they were excluded. Finally, 31 patients had GN and were further genetically analyzed.

Patients’ whole blood was sampled for WES. Detailed data on the sociodemographic and clinical characteristics of GN patients were collected. We used the GnomAD data to compare allele frequencies between our cohort and a large cohort of the general population. We have already had a cohort of 50 nephrologically and immunologically healthy pediatric patients undergoing WES because of the isolated foot deformities. We considered that this cohort may be important as a control group because it included patients from Serbia. Like our GN group, all patients were children (which is not the case with the GnomAD database), and all patients were tested in the same laboratory (3billion) using the same methodology and data interpretation. Thus, this cohort served as a control group (CG). They were analyzed for the presence of the variants detected in the GN cohort. Out of 50 CG patients tested, 31 (62%) were males, while 19 (38%) were females. The average age of these patients was 8.8 ± 4.3 years.

### 2.2 Molecular genetic analysis

WES was performed at the reference laboratory for genetic testing, 3billion, Inc., Seoul, South Korea, using the xGen Exome Research Panel v2 supplemented with the xGen Human mtDNA Panel for Mitochondrial DNA and the xGen Custom Hyb Panel v1 (Integrated DNA Technologies, Coralville, United States) on the Illumina NovaSeq 6000 platform (Illumina, San Diego, United States). The analysis and interpretation of the obtained data consisted of the alignment to the GRCh37/hg19 human reference genome and variant calling, which were done using open-source bioinformatic tools. Annotation, filtering, and classification of variants were done with the *in-house* software EVIDENCE (Seo et al., 2020). The frequency of certain variants in other populations and globally was assessed using the gnomAD project (http://gnomad.broadinstitute.org/) (Karczewski et al., 2020). Data on the pathogenicity of a given variant and its association with a certain disease were retrieved from databases such as OMIM (www.omim.org), ClinVar, and UniProt (Hamosh et al., 2005; Landrum et al., 2020). Prediction of the functional effect of each variant and the degree of evolutionary conservation was evaluated using *in silico* prediction software (listed in Table 2). The pathogenicity of each variant was assessed according to the American College of Medical Genetics and the Association for Molecular Pathology (ACMG/AMP) recommendations (Richards et al., 2015). The clinical phenotype of each patient was translated into the corresponding human phenotype ontology (HPO) terms (https://hpo.jax.org/) (Köhler et al., 2021) and used to calculate similarity to each of approximately 7,000 rare genetic diseases (Greene et al., 2016; Köhler et al., 2009). Each patient’s symptoms were compared to every known symptom of the given disease.

Finally, the EVIDENCE software selected as priority variants classified as pathogenic, likely pathogenic, and variants of uncertain significance (VUS) according to ACMG/AMP recommendations. They are categorized into a three-level system based on the Bayesian score (Tavtigian et al., 2018). The first degree is variants with a score over 0.9, the second degree with a score over 0.499, and the third with a score over 0.1. All rare variants detected in genes associated with specific diseases were further considered to be characterized by medical geneticists. Variants in genes with a lower similarity score with the symptoms of a given patient were manually evaluated. In addition, rare variants that were not in the genes associated with the symptoms given for a particular patient were analyzed.

All high-priority variants detected in the GN cohort were screened in the CG group to estimate variant frequency in this population. A BaseSpace Variant Interpreter (Illumina, San Diego, United States) was used for this purpose.

### 2.3 Statistics

Descriptive statistics methods were used: proportion, median, and interquartile range.

## 3 Results

Sociodemographic and clinical data of the investigated cohort are presented in Table 1. The study comprised 10 patients with primary glomerulonephritis (five with IgAN, two with SSNS, one with FSGS, one with MGN, and one with anti-GBM disease), 14 patients with LN, and seven with HSP nephritis.

**TABLE 1:** Sociodemographic and clinical features of investigated patients.

Feature	All	Primary GN	SLE and GN	HSP and GN
N	31	10	14	7
Gender (% males)	51.6%	80.0%	21.4%	71.4%
Age at onset (years, median (IQR))	11 (5–14)	9.5 (4–13)	13 (11–15.5)	5 (4–7)
Age at sampling (years, median (IQR))	15 (8–17)	15 (10–16)	17 (15–18)	7 (4–8)
Disease duration (months, median (IQR))	28 (13–53)	39 (30–78)	22.5 (11–61)	4 (2–22)
Therapy (%)				
None	6.4%	20.0%	0.0%	0.0%
CS	29.0%	30.0%	7.1%	71.4%
CS and IS	54.8%	30.0%	85.7%	28.6%
CS, IS, and mab	9.7%	20.0%	7.1%	0.0%
Number of GN relapses	1 (0–3)	2 (0.5–4.5)	1 (1–2)	0 (0–2.5)

CS, corticosteroids; GN, glomerulonephritis; HSP, Henoch–Schoenlein purpura; IQR, interquartile range; IS, immunosuppressants; mab, monoclonal antibodies; SLE, systemic lupus erythematosus.

Results are presented as proportions or medians (interquartile range).

Among 10 patients with primary glomerulonephritis, two patients had positive findings on WES (20%). Detailed variant analysis is presented in Table 2.

**TABLE 2:** GN cases with high-priority variants identified by WES.

Gene	Patient no.	Clinical diagnosis	Nucleotide change	Protein change	Variation type, molecular consequence, variant location	Clinical significance (ACMG categories)	GnomAD exome v4//gnomAD genomes v4 MAF	Cohort of 50 healthy pediatric controls	Theoretical predictions	Inheritance/zygosity	Reference/novel variant	Disease (OMIM)
*COL4A3*	G034	Primary GN	NM_000091.5:c.3546_3548dup	p. (Gly1183dup)	Duplication, in-frame, exon	LP (PS1, PM1,PM2, PS4:Supporting, PM4:Supporting)	0.00000205//0.00000657	Not found	NA	AD/heterozygous	ClinVarID:438655; PMID:30586318	Alport syndrome type 3A (OMIM:104200)
*CD46*	G026	Primary GN	NM_172358.3:c.1122G>C	p. (Ter374Tyrext*23)	SNV, stop-loss, exon	VUS (PM4, PM2, BP4)	Not found//not found	Not found	EIGEN PC: benign strong; FATHMM-MKL: benign strong; DANN: benign moderate; MutationTaster: uncertain; EIGEN: benign moderate	AD/AR/heterozygous	novel	Atypical hemolytic uremic syndrome type 2 (aHUS) (OMIM:612922)
*C2*	G032, G027	Two with SLE and GN	NM_000063.6:c.841_849 + 19del	p.?	Deletion, splice junction loss, intron	P (PVS1, PS3, PP1, PP5, BS2)	0.0046 heterozygotes/0.000008 homozygotes//0.00608 heterozygotes/0.000032 homozygotes	One control-0.02	NA	AR/heterozygous	ClinVar ID:50,634; PMID:35874679; 34426522; 33726816, 31980526; 31440263; 29619023; 27943079; 26590091; 26038300; 25454804; 11115162; 9616367; 8645999; 7901282; 1577763	C2 deficiency (OMIM:217000)

AD, autosomal dominant; AR, autosomal recessive; BP, benign supporting; BS, benign strong; LP, likely pathogenic; NA, not available; P, pathogenic; PM, moderate evidence of pathogenicity; PP, supporting evidence of pathogenicity; PS, strong evidence of pathogenicity; PVS, very strong evidence of pathogenicity; SNV, single nucleotide variant; VUS, variant of uncertain significance.

We detected one likely pathogenic heterozygous in-frame variant (c.3546_3548dup, p. (Gly1183dup)) in the *COL4A3* gene in one patient that was absent in our CG. Variants in *COL4A3* are associated with autosomal dominant Alport syndrome type 3A (OMIM:104200), autosomal recessive Alport syndrome type 3B (OMIM:620536), and autosomal dominant familial benign hematuria type 2 (OMIM:620320). A c.3546_3548dup variant has been previously described in patients with autosomal dominant Alport syndrome (Groopman et al., 2019). In our patient, symptoms started at the age of six with microhematuria. At that time, the histopathology of the kidney was inconclusive. At the age of 9, he developed proteinuria, and at the age of 10, he developed bilateral sensorineural hearing impairment for high frequencies, which is all consistent with the diagnosis of Alport syndrome. His mother had the same mutation and was asymptomatic.

A heterozygous stop-loss novel variant of uncertain significance (c.1122G>C, p. (Ter374TyrextTer23)) was identified in the *CD46* gene in one patient with primary GN. Variants in *CD46* are associated with susceptibility to atypical hemolytic uremic syndrome (aHUS) type 2 (OMIM:612922), which can be inherited in an autosomal dominant or recessive manner. The patient’s symptoms started at age 18, with hemoptysis, seizures, microhematuria, and anemia. He was later positive for anti-GBM antibodies, having a diagnosis of Goodpasture syndrome. He was treated with corticosteroids and cyclophosphamide, with a positive response.

In two of 14 patients with LN, a heterozygous pathogenic variant (c.841_849 + 19del, p.?) was detected in the *C2* gene. Their disease onset was earlier than in other patients with lupus nephritis (10 years vs 13.5 years). Their response to steroid and immunosuppressive therapy was good. This variant was also detected in a heterozygous state in one case from our CG. This patient had no symptoms or signs of any autoimmune disease. Variants in *C2* are associated with autosomal recessive C2 deficiency (OMIM:217000). A c.841_849 + 19del variant has been previously described in homozygous and compound heterozygous states in patients with complement C2 deficiency (Mørup et al., 2022; Kars et al., 2021; Stranneheim et al., 2021; Hou et al., 2020). It involves the deletion of a 5′donor splice site and is subsequently predicted to alter proteins.

We found no significant variants on WES in seven patients with HSP and GN.

## 4 Discussion

Genetic analysis in pediatric patients with GN may be important for several reasons. In the first place, hereditary diseases that have a clinical picture like GN and require specific monitoring and treatment, such as aHUS and Alport syndrome, can be detected. Second, finding susceptibility genes in GN may help understand disease pathophysiology and offer new therapies to patients.

Two of our 10 patients with primary GN had a genetic diagnosis of another disease. One likely pathogenic heterozygous in-frame variant (c.3546_3548dup) was identified in the *COL4A3* gene in our patient, primarily diagnosed with IgA nephropathy that was not confirmed by histopathology. Variants in *COL4A3* are associated with autosomal dominant Alport syndrome type 3A (OMIM:104200), autosomal recessive Alport syndrome type 3B (OMIM:620536), and autosomal dominant familial benign hematuria type 2 (OMIM:620320). Our patient eventually developed the full presentation of Alport syndrome. A c.3546_3548dup variant has been previously described in a heterozygous state in patients with Alport syndrome (Groopman et al., 2019). This variant is present at a frequency of 0.00000205 and 0.00000657 in gnomAD exomes and genomes, respectively, and was not found in our 50 pediatric controls. The patient’s mother had the same mutation and was asymptomatic. It is possible that the hypomorphic allele, modifying variants in some other genes, or the presence of a deep intronic variant in the second allele not visible by WES may explain the phenotype in our patient. The coexistence of Alport syndrome and Fabry disease in a patient with IgA nephropathy has been reported (Hao et al., 2020). As stated by Rahimzadeh and colleagues in their case report, a high rate of Alport misdiagnoses has been seen due to the rarity of this disease, particularly in cases without extrarenal manifestations (Rahimzadeh et al., 2023). In this case, genetic findings provide new clinical insight, have the potential to initiate multidisciplinary care, and may influence the choice of therapy (Groopman et al., 2019; Canetta and Radhakrishnan, 2015).

We have detected one heterozygous stop-loss novel variant of uncertain significance (c.1122G>C) in the *CD46* (*MCP*) gene in one patient with primary GN–anti-GBM disease. The described variant is absent from gnomAD and our 50 CG cohort, which suggests its pathogenicity, but we were not able to perform segregation analysis in the family because parents did not want to give their blood samples. Also, we were not able to conduct functional analysis due to technical reasons. Thus, we cannot claim that this variant is pathogenic. Variants in *CD46* are associated with susceptibility to aHUS type 2 (OMIM:612922), which can be inherited in an autosomal dominant or recessive manner. Genetic variants in CD46 have been previously associated with other diseases such as SLE, systemic sclerosis, miscarriage, and glomerulonephritis (Jönsen et al., 2011; Mohlin et al., 2013; Scambi et al., 2015; Servais et al., 2007). A previous study analyzing 248 patients with different biopsy-proven glomerulopathy discovered that six patients developed aHUS (Manenti et al., 2013). However, reports of an association of CD46 variants and anti-GBM disease have not yet been described, to our knowledge. Variants in aHUS are predominantly heterozygous; however, rare homozygous variants were associated with earlier onset and more severe symptoms (Caprioli et al., 2006). One study found that approximately 10% of patients with aHUS (11 patients of 120) have variants in *MCP*, of which five were heterozygous variants that resulted in reduced levels of MCP. Furthermore, incomplete penetrance was noticed in those patients, suggesting that *MCP* is rather a predisposing factor than a direct cause (Esparza-Gordillo et al., 2006; Fremeaux-Bacchi et al., 2006).

A heterozygous pathogenic variant (c.841_849 + 19del) has been detected in the *C2* gene in two patients with LN, as well as in one of our 50 patients from the CG group. Variants in *C2* are associated with autosomal recessive C2 deficiency (OMIM:217000) (Mørup et al., 2022; Kars et al., 2021; Stranneheim et al., 2021; Hou et al., 2020). Our variant has been found with a frequency of 0.0046 in gnomAD exomes, and 0.000008 were homozygotes, while in gnomAD genomes, it was found with a frequency of 0.00608, and 0.000032 were homozygotes. A higher frequency was estimated in our CG cohort, where it was detected in 2% of individuals. Patients with heterozygous c.841_849 + 19del have been described in the literature. One was a girl with SLE and significant nephritis within 2 years after onset who underwent renal transplantation, and the second was a boy with active arthritis (Spârchez et al., 2015). Furthermore, a patient with symptoms that started in her twenties was described as having a heterozygous c.841_849 + 19del variant with a pathogen-specific immunodeficiency (Mørup et al., 2022).

The main limitation of the study is the small number of patients and a heterogeneous cohort of different GNs. It is also notable that the number of patients with primary GN was only 10. Thus, the positive result rate of WES of 20% may be exaggerated due to a selection bias. However, our aim was to provide additional information about a pediatric population that is underrepresented in the literature and to give the first insight into the specific genetic background of this condition in the Balkans. We understand that further functional studies of identified variants, especially the novel variant in the *C2* gene, will be of interest, but we were not able to perform them.

## 5 Conclusion

In this study, we have analyzed WES data of 31 sporadic pediatric patients with GN. We detected one likely pathogenic heterozygous in-frame variant in the *COL4A3* gene associated with Alport syndrome in one patient originally diagnosed with primary GN. A heterozygous stop-loss novel variant of uncertain significance was found in the *CD46* gene associated with aHUS in a patient with primary GN. A heterozygous pathogenic variant (c.841_849 + 19del, p.?) in the *C2* gene was found in two patients with SLE and GN. This analysis helped us detect hereditary diseases that have a clinical picture similar to GN. Finding susceptibility genes in various GNs may help understand disease pathophysiology and offer new therapies to patients.

## Data Availability

The datasets for this article are not publicly available due to concerns regarding participant/patient anonymity. Request to access the datasets should be directed to the corresponding author.
